# Transcriptomic Analysis Suggests Genes Expressed Stage-Independently and Stage–Dependently Modulating the Wing Dimorphism of the Brown Planthopper

**DOI:** 10.3390/genes11010019

**Published:** 2019-12-23

**Authors:** Chao Zhang, Xiang-Dong Liu

**Affiliations:** Department of Entomology, Nanjing Agricultural University, Nanjing 210095, China; 2013202048@njau.edu.cn

**Keywords:** developmental stage, genetic determination, gene expression, *Nilaparvata lugens*, transcriptome, wing morph

## Abstract

Wing dimorphism is considered as an adaptive trait of insects. Brown planthoppers (BPHs) *Nilaparvata lugens*, a serious pest of rice, are either macropterous or brachypterous. Genetic and environmental factors are both likely to control wing morph determination in BPHs, but the hereditary law and genes network are still unknown. Here, we investigated changes in gene expression levels between macropterous and brachypterous BPHs by creating artificially bred morphotype lines. The nearly pure-bred strains of macropterous and brachypterous BPHs were established, and their transcriptomes and gene expression levels were compared. Over ten-thousand differentially expressed genes (DEGs) between macropterous and brachypterous strains were found in the egg, nymph, and adult stages, and the three stages shared 6523 DEGs. The regulation of actin cytoskeleton, focal adhesion, tight junction, and adherens junction pathways were consistently enriched with DEGs across the three stages, whereas insulin signaling pathway, metabolic pathways, vascular smooth muscle contraction, platelet activation, oxytocin signaling pathway, sugar metabolism, and glycolysis/gluconeogenesis were significantly enriched by DEGs in a specific stage. Gene expression trend profiles across three stages were different between the two strains. Eggs, nymphs, and adults from the macropterous strain were distinguishable from the brachypterous based on gene expression levels, and genes that were related to wing morphs were differentially expressed between wing strains or strain × stage. A proposed mode based on genes and environments to modulate the wing dimorphism of BPHs was provided.

## 1. Introduction

Polyphenism is a life history strategy for organisms to deal with heterogeneous environments. Two or more distinct morphs can arise from a genotype as a result of differing environmental conditions [1]. Wing dimorphism provides an opportunity for insects to choose migration or settlement. An aphid clone can produce both the wingless and winged offspring under different conditions, such as population density [2,3], and a pair of rice planthopper parents can generate two kinds of progeny with long or short wings [4,5]. The waterstrider *Limnoporus canaliculatus* can produce both the long-winged and wingless morphs that are determined by genetic component and photoperiod [6]. It seems that the wing dimorphism in insects is a phenotypic plasticity of a morphological trait, but it has been confirmed in multiple species that the wing morph is solely determined by genetic mechanisms, or solely by environmental mechanisms, or through a combination of both [7,8,9]. In *Myzus persicae*, mitochondrial adenine nucleotide translocase (ANT), the chemoreception and takeout-like (TOL) genes showed higher expression levels in the winged morphs than in the wingless morphs [10]. The genetic and molecular mechanisms underlying wing dimorphism in insects are attracting more attention in the last decade, due to the development of molecular techniques [7,10,11]. However, the genetic law and genes network determining wing polymorphism are still unclear.

The rice brown planthopper (BPH), *Nilaparvata lugens*, which is a devastating pest of rice, displays an obvious wing dimorphism, and it is a better model organism to study on the wing morph determination [7]. In a wild population, the long- and short-winged morphs often coexist [12]. Previous researches have shown that genetic and environmental factors together contribute to the wing morph of rice planthoppers [5,13,14]. The nutrient of host plants, population density, and climate are all factors that are known to affect wing morphs of insects [13,14,15,16,17,18,19,20]. Phenotypic differentiation of wing morphs might be triggered by environment cues occurring in some specific developmental stages of insects [21,22,23,24,25]. Wing induction in aphids can be controlled either by the mother (pre-natal) or by the developing nymph (post-natal) crowding, depending on aphid species [23,26]. The 3rd or 4th-instar nymphs of BPHs are the most sensitive to a decrease of population density, which induces the short-winged morph [22,24]. The determination of wing morphs, long- or short-winged, and winged or wingless in insects might be made in a short window period before adult emergence, and this period is generally named the ‘sensitive stage’. These results imply that gene roles in determining wing morphs might be various in different developmental stages of insects. Therefore, it is worth studying changes in the gene expression levels between the long- and short-winged insect strains across all developmental stages to highlight the molecular mechanism underlying wing dimorphism.

Gene expression differences between the winged and wingless, or long- and short-winged morphs have been illustrated in some insects [27,28]. More than one-thousand of differentially expressed genes were identified between two wing morphotypes [27,28,29]. For example, 1663 differentially expressed transcripts were identified from the winged and wingless cotton aphids *Aphis gossypii*, while using a tag-based digital gene expression approach, and these transcripts were enriched in the metabolic pathways of ribosome, pyruvate metabolism and proteasome, protein synthesis and degradation, lipid metabolism, immunity, RNA transport, and some signaling pathways [28]. In 1734 unique cDNAs (an estimated 10% of coding genes) from the pea aphid *Acyrthosiphon pisum*, there were 141 and 142 genes with differential transcript accumulation between winged and wingless morphs in the fourth instar nymph and adult, respectively, and the functions of differentially accumulated transcripts mainly enriched in muscles and energy production [27]. Six out of 11 wing development-related genes showed significant differences in expression level among five developmental stages of the pea aphid, and another two genes exhibited a significant development stage × wing morph interactive effect [30]. In the New Zealand stonefly *Zelandoperla fenestrata*, the fully winged and vestigial-winged morphotypes strongly differentiated in small regions of the genome [31]; nine and one wing development-related gene clusters were significantly upregulated in full-winged and vestigial-winged ecotypes, respectively [32]. In the third-instar nymphs of BPHs, there were 2544 differentially expressed genes between nymphs that were reared on yellow-ripe stage of rice with a high population density (inducing long-winged morphs) and on tillering stage rice with a low population density (inducing short-winged morphs), and the expression levels were dependent on the developmental stage of nymphs [29,33]. The molecular mechanism underlying wing polymorphism in insects is still not clear [34], although a previous study has known that the expression of two insulin receptors (*InR1* and *InR2*) determined wing morphs of rice planthoppers [7].

Interactions between genetics and environment conditions manipulate the wing morph of rice planthoppers [4,18,35,36,37]. The direct selection for wing morphs in BPHs under a high-density condition was successful in obtaining the long-winged and short-winged pure-bred lines [38]. Therefore, using the pure-bred lines to study on variations in transcriptome and gene expression levels between the two wing morphotypes would be much better than using the wild populations to evaluate the genes network in determining the wing morphs of insects. In this study, we hypothesized that genetic pathways that enriched differentially expressed genes globally or stage-dependently across egg, nymph and adult stages determined the wing dimorphism of BPHs. Therefore, the long- and short-winged pure-bred or nearly pure-bred strains of BPHs were set up while using the successively directional selection, and then transcriptomes of their eggs, 3rd-instar nymphs and adults were sequenced. The differentially expressed genes and their enrichment pathways between the two strains and among the three growth stages were analyzed. The results will provide more evidence for the genetic and molecular mechanisms of wing dimorphism in insects.

## 2. Materials and Methods

### 2.1. Insects

The brown planthoppers (BPHs), *Nilaparvata lugens*, were collected from rice field in Nanjing, China and reared in a chamber under a 14 h light:10 h dark photoperiod at 25 ± 1 °C and a relative humidity of 75% ± 10% using rice seedlings (rice variety: Wuyunjing-7) [5,14]. The newly born long-winged and short-winged adults were used in the selection experiments for wing morphs.

### 2.2. Selection of Macropterous and Brachypterous Pure-Bred Strains

**Figure 1:**
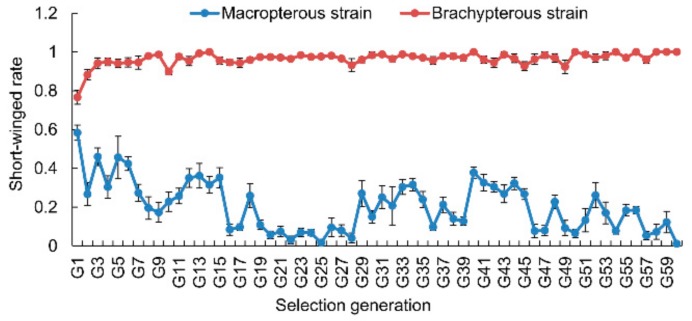
Short-winged rate in the macropterous and brachypterous strains of brown planthoppers (BPHs) in different selection generations.

The long-winged (macropterous) and short-winged (brachypterous) strains were established through sibling inbreeding for successive generations under a constant condition [5]. In each selection generation, 10 pairs of adults and 30 first-instar nymphs from each pair of adults were examined. During a selection process, a pair of newly emerged unmated female and male adults with the same wing form were paired (M♀ × M♂ or B♀ × B♂), copulated and produced progenies in a plastic cup (diameter 80 mm, height 100 mm) with 10 rice seedlings. Ten independent pairs were set up for each selected lineage. Within 24 h after the eggs hatching, 10 first-instar nymphs were collected and reared in a plastic cup with 10 rice seedlings, and three replications of nymphs were performed for each pair of adults. In each generation, the wing form of all emerged adults was examined and the short-winged morph rate was calculated. The offspring adults with an identical wing form as their parents were selected for sibling mating to produce the next generation. After 38 and 36 successive generations of selection in the long-winged and short-winged lineages, respectively, the percentages of the short-winged adults were 13.98% ± 3.33% and 95.67% ± 2.01% (Figure 1). These long-winged and short-winged lineages were considered as the nearly pure-bred macropterous (M) and brachypterous (B) strains, and their eggs (named ME and BE), 3rd-instar nymphs (M3rd and B3rd), and adults (MA and BA) were used for the transcriptome sequencing and qPCR experiments. Additionally, the selection of wing morphs remained.

### 2.3. RNA Extraction and cDNA Library Construction for Transcriptome Sequencing

The samples used for transcriptome sequencing were collected from the 39th generation of macropterous strain and 37th generation of brachypterous strain. The eggs, third-instar nymphs, and adults from the macropterous and brachypterous strains were examined. Adults from the 38th generation-selected macropterous strain and the 36th generation-selected brachypterous strain were chosen to produce eggs on rice seedlings. The seedlings were renewed at a 24 h interval and dissected seedling tissue to collect eggs five days later, and these fertilized eggs with red eyepoints were dissected out from rice leaf sheaths while using a pair of ophthalmic forceps under a dissection microscope. 900 eggs were collected for extracting RNA. 40 nymphs were collected when the nymphs grew up to the 3rd instar. When adults emerged, the 24–36 h-old adults (15 females and 15 males) were collected and pooled as an adult sample for transcriptome sequencing. All of the samples were frozen in liquid nitrogen and then stored at −80 °C.

Total RNA was isolated while using TRIzol reagent (Invitrogen, Carlsbad, CA, USA) and treated with DNase I (Takara, Dalian, China), according to the manufacturer protocol. The purity and integrity of total RNA were determined by spectrophotometer NanoDrop ND-8000 (Thermo Fisher, Waltham, MA, USA) and Agilent 2100 Bioanalyzer (Agilent, Santa Clara, CA, USA). The samples with RNA integrity number (RIN) > 8.0 were used for sequencing. More than 20 μg RNA for each sample was obtained to construct the cDNA library.

### 2.4. cDNA Library Construction and Sequencing

Six cDNA libraries were separately constructed according to the TruSeq RNA Sample Prep Kit v2 (Illumina, San Diego, CA, USA). 200 ng total RNA sample was purified by oligo-dT magnetic beads, and then poly (A)-containing mRNA were fragmented into small pieces with Elute, Prime, Fragment Mix. The first-strand cDNA was synthesized while using First Strand Mater Mix and Super Script II (Invitrogen, Carlsbad, CA, USA) reverse transcription (The reaction condition: 25 °C for 10 min.; 42 °C for 50 min.; 70 °C for 15 min.), and then added the Second Strand Master Mix to generate the second-strand cDNA (16 °C for 1 h). The purified fragmented cDNA was combined with the End Repair Mix and then incubated at 30 °C for 30 min. The end-repaired DNA was purified with Ampure XP Beads (Agencourt, Danvers, MA, USA) and then added the A-Tailing Mix and mixed well by pipetting and incubated at 37 °C for 30 min. The cDNA fragments with Poly (A) addition was combined with the Adenylate 3′ Ends DNA, RNA Index Adapter and Ligation Mix, and then mixed well by pipetting to connect the sequencing adaptor. The ligate reaction was incubated at 30 °C for 10 min. The end-repaired DNA was purified with Ampure XP Beads, and then several rounds of PCR amplification with the PCR Primer Cocktail and PCR Master Mix were performed to enrich the cDNA fragments. The PCR products were purified with Ampure XP Beads and created the final cDNA library. The qualified library was amplified on cBot to generate the cluster on the flowcell (TruSeq PE Cluster Kit V3-cBot-HS, Illumina), and the amplified flowcell was sequenced pair end on the HiSeq2000 System (TruSeq SBS KIT-HS V3, Illumina) at Beijing Genomics Institute (Shenzhen, China). The read length was 90 bp.

### 2.5. De novo Assembly and Annotation

Based on the eggs, third-instar nymphs and adults from both the macropterous and brachypterous strains, 2.8 × 10^10^ bases in total were generated in all six transcriptomes. The clean reads were obtained via removing reads with adaptors and unknown nucleotides larger than 5%, and low-quality reads (the rate of bases with quality value ≤ 10 was more than 20%). The proportions of unknown nucleotides in clean reads were 0.01% for all six samples. Transcriptome de novo assembly was carried out while using these short clean reads on Trinity program (version v2.0.6) and attained the unigenes. Subsequently, the unigenes were taken into further process of sequence splicing, redundancy removing and clustering with TGICL tools version 2.1 [39,40]. Unigene sequences were firstly aligned to protein databases NR, Swiss-Prot, Kyoto Encyclopedia of Genes and Genomes (KEGG) and Clusters of Orthologous Groups (COG) (*e*-value < 0.00001) by Blastx (version v2.2.23), and nucleotide database NT (*e*-value < 0.00001) by Blastn (version v2.2.23), retrieving proteins with the highest sequence similarity with the given unigenes, along with their protein functional annotations. The sequence direction and the coding regions of unigenes were determined according to the best aligning results. The ESTScan program predicted unigenes unaligned to none of the above databases [41]. The unigenes were mapped to the COG database and predicted the possible functions. With NR annotation, Blast2GO program (version v2.5.0) was used to get Gene Ontology (GO) annotation of unigenes. WEGO software [42,43] was used to undertake GO functional classification for all unigenes and understand the distribution of gene functions of the species from the macro level after obtaining GO annotation for every unigene. With the help of the KEGG database, genes biological complex behaviors were further studied, and using KEGG annotation we obtained pathway annotation for unigenes. InterProScan5 performed the InterPro annotation (version v5.11-51.0).

### 2.6. Analysis of Differential Gene Expression Profiles

Bowtie2 remapped clean reads to the unigenes (version v2.2.5). The relative expression levels of all the matched unigenes were normalized by transforming the clean data to fragments per kilobases of transcripts per million mapped fragments (FPKM) by RSEM (version v1.2.12). The log_2_(fold change) of FPKM of an unigene in one transcriptome to another was used to determine the differentially expressed gene (DEG). The DEGs between the macropterous (M) and brachypterous (B) strains in the egg, 3rd-instar nymph, and adult stages were identified based on the Poisson distribution method [44]. False discovery rate (FDR) was calculated to determine the threshold *p*-value in multiple test. Transcripts with a minimal two-fold change in expression (|log_2_ fold change| ≥ 1) and FDR ≤ 0.001 were considered as DEGs between two samples.

GO and KEGG pathway enrichment of DEGs were performed. This enrichment analysis would find the main biological functions of DEGs, and the main biochemical pathways and signal transduction pathways that DEGs took part in. The enriched *p*-values were calculated according to the hypergeometric test and performed with Bonferroni correction. The GO terms with corrected *p*-value < 0.05 were defined as significantly enriched GO terms in DEGs. The REVIGO web tool was used to summarize the long lists of GO terms with the default parameters in order to remove redundant GO terms [45]. For the pathway enrichment, the multiple testing correction *Q* value < 0.05 was used as the threshold.

The principal component analysis (PCA) was performed for the six samples (ME, M3rd, MA, BE, B3rd, and BA) based on the FPKM of each unigene after data standardization while using the z-score method. The first two principal components (PC1 and PC2) interpreted 64.9% of variances. Therefore, we used the PC1 and PC2 to distinguish the six samples (egg, 3rd-instar nymph, and adult from the macropterous and brachypterous strains). The hierarchical clustering of six samples was analyzed based on the expression levels of 6523 DEGs shared with eggs, nymphs, and adults.

Expression pattern analysis (trend analysis) was performed to obtain the transcriptional differences over the developmental stages between the macropterous and brachypterous strains. All DEGs across three developmental stages were assigned to eight expression profiles while using a short time-series expression miner STEM (version v1.3.8). The unigenes belonging to the same expression profiles had similar expression pattern among the growth stages. For each strain, the clustered profiles with *p* < 0.05 were considered as the significant trend profiles. GO and KEGG enrichment were performed in the trend profiles.

### 2.7. Expression Levels of Genes Related to Wing Development in Macropterous and Brachypterous Strains

Eight differentially expressed transcripts that were related to the wing development of insects [7,30,46] were selected for measuring their expression levels in the macropterous and brachypterous strains during the egg, 3rd instar nymph, and adult stages while using the qPCR method (Table 1). The total RNA was extracted from eggs, third-instar nymphs, female adults, and male adults from the macropterous and brachypterous strains. A total of 1000 ng total RNA from each sample was used to synthesize the single-strand cDNA while using PrimeScript RT reagent kit with gDNA Eraser (TAKARA, Dalian, China). 18S rRNA gene was used as an internal reference gene. Gene-specific primers were designed with Beacon Designer (version 7.0) and Oligo (version 7.0) software (Table 1), and the primer specificity was verified while using the dissociation curve analysis. The qPCR was performed using SYBR Premix Ex Taq kit (TAKARA, Dalian, China) according to the manufacture’s protocol in an ABI 7500 (Applied Biosystems, Carlsbad, CA, USA). Each reaction mixture was 20 μL containing 10 μL SYBR Premix Ex Taq, 200 nM of each forward and reverse primer, 0.4 μL ROX reference DyeⅡ. The cycling parameters were 95 °C for 30 s, followed by 40 cycles of 95 °C for 5 s, 60 °C for 34 s. All of the reactions were performed in triplicate, and dissociation curve analysis was performed after each assay to determine the target specificity. The cycle threshold (Ct) was normalized to the 18S rRNA gene (ΔCt). The Ct values of 18S rRNA gene were stable between the macropterous (14.45 ± 0.20) and brachypterous (14.62 ± 0.22) strains. The ΔCt of the macropterous sample was calibrated against the corresponding brachypterous sample to obtain the ΔΔCt value, and this value was used to evaluate the fold-change of gene expression level in the macropterous strain related to the brachypterous strain. The ΔCt value from a sample was calibrated again with one sample from a specific stage of BPHs and obtained another ΔΔCt, and the 2^−ΔΔCt^ was used to calculate the relative expression level of a gene in a sample. Three biological replications were performed for all of the experiments. The correlation of the fold changes of eight genes between the macropterous and brachypterous strains measured by the RNA-seq and qPCR was analyzed while using the Pearson method. The maximum of the fold change from a female sample and a male sample measured by qPCR was considered as the fold change of an adult sample because females and males were pooled in an adult sample for the RNA-seq. The effects of the BPH strain and developmental stage on the fold changes were analyzed while using GLM, and the differences in expression levels of each gene between the macropterous and brachypterous strains were analyzed using student t-test. All of the statistics were performed in IBM SPSS Statistics 25.

## 3. Results

### 3.1. Selection Response of Wing Morphs

The selection response of BPHs in wing morphs was strong (Figure 1). The frequency of the short-winged morph increased up to 92–100% and maintained during 3–60 generations of directional selection in the brachypterous female × brachypterous male lineage. The frequency of the long-winged morph could increase up to approximately 95% after 20 generations of directional selection in the macropterous female × macropterous male lineage, nevertheless the frequency still fluctuated during 29–60 generations (Figure 1).

### 3.2. Differentially Expressed Genes between the Macropterous and Brachypterous Strains

**Figure 2:**
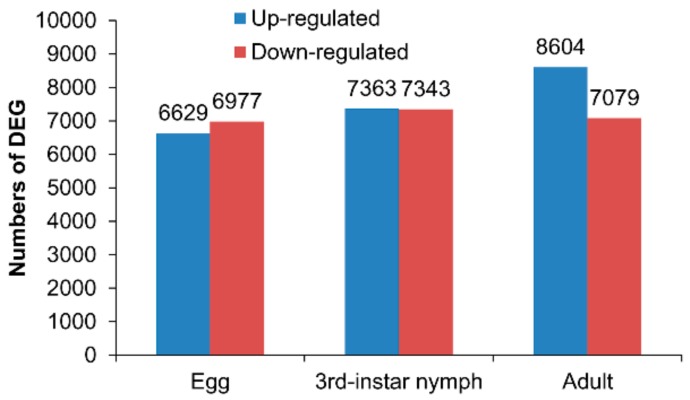
Differentially expressed genes (DEGs) of the macropterous strain in eggs, 3rd instar nymphs, and adults, when compared to the brachypterous strain.

Illumina sequencing generated 54.42–57.60 million of 90 bp pair-end raw reads from the six samples (NCBI SRA accession: SRR10008513), and the total mapped reads were 93.03–94.49%. In the results of assembly, 77,765 unigenes were detected (Table 2). The total length for unigenes was 102,867,302 nt with 1323 nt of average length and 2482 nt of N50. The total annotation unigenes were 34,589. The wing dimorphism resulted in expression differentiation of many genes in BPHs across three growth stages. As shown in Supplementary Materials Tables S1–S3, 13,606, 14,706, and 15,683, DEGs were identified between the macropterous and brachypterous strains in the eggs, 3rd instar nymphs, and adults, respectively, accounting for 17.5, 18.9, and 20.2 percent of total unigenes (Figure 2).

The total number of DEGs increased as the development of brown planthoppers from egg to adult. In eggs and third instar nymphs, nearly the same numbers of genes were upregulated vs. downregulated in the macropterous strain as compared to the brachypterous strain (Figure 2). In contrast, substantially more genes were upregulated in macropterous adults as compared to brachypterous adults (Figure 2).

### 3.3. GO Term and KEGG Classification for DEGs between the Macropterous and Brachypterous Strains

**Figure 3:**
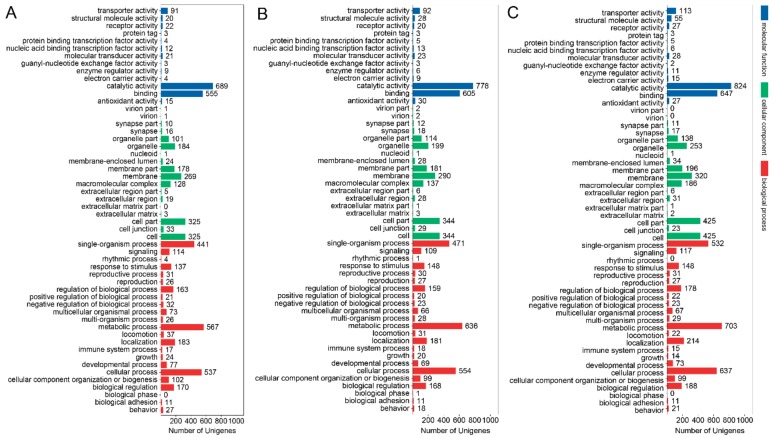
Gene ontology (GO) classification for DEGs between the macropterous and brachypterous strains in the growth stages of egg (**A**), 3rd instar nymph (**B**), and adult (**C**).

The GO term showed that the DEGs between the macropterous and brachypterous strains were distributed across the similar biological process, cellular components, and molecular functions across the three different growth stages of BPHs egg, nymph, and adult (Figure 3).

While using the Revigo web tool, we found that significantly enriched GO terms were mainly involved in cell fate determination, regulation of cell projection organization, site of polarized growth, growth cone, microtubule binding, and phosphate transmembrane transporter activity in eggs, whereas significantly enriched GO terms were the metabolic process, catalytic activity, and oxidoreductase activity, etc. in the 3rd-instar nymphs and adults (Table 3).

**Figure 4:**
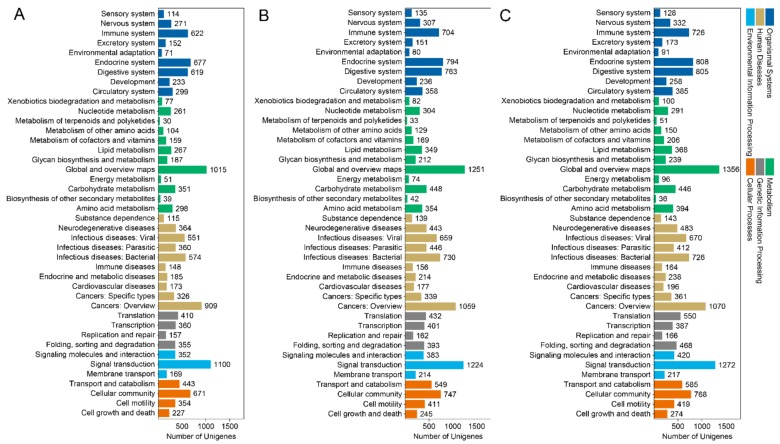
Kyoto Encyclopedia of Genes and Genomes (KEGG) pathway classification of DEGs between the macropterous and brachypterous strains in egg (**A**), 3rd instar nymph (**B**), and adult (**C**) growth stages.

Similarly, the numbers of DEGs between the macropterous and brachypterous strains were classified into similar KEGG pathways among the three growth stages of BPHs. Overall, many DEGs were involved in metabolism, environmental signal transduction, bacterial infection, endocrine, and digestive systems (Figure 4).

**Figure 5:**
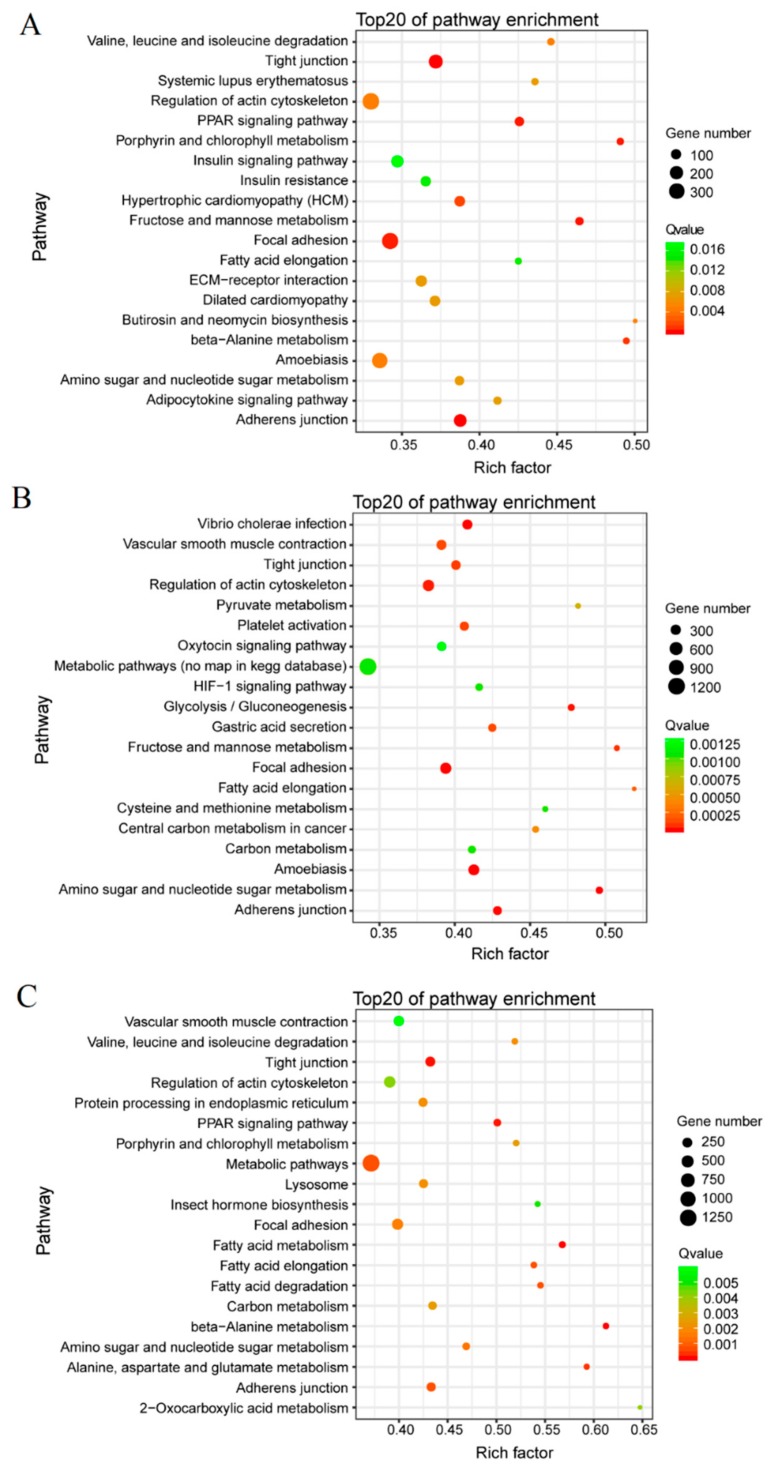
Top 20 enrichment pathways for DEGs between the macropterous and brachypterous strains in the egg (**A**), 3rd instar nymph (**B**), and adult stages (**C**). The *Q*-values are shown by different color, and less than 0.05 denotes the significant enrichment. Different sizes of dots mean the gene number enriched in a pathway.

The DEGs were significantly enriched in four KEGG pathways: regulation of actin cytoskeleton, focal adhesion, tight junction, and adherens junction across three growth stages of BPHs: egg, nymph, and adult (Figure 5). Additionally, the insulin signaling pathway and amoebiasis were enriched with DEGs in the egg stage (Figure 5A), and many DEGs were enriched in metabolic pathways, amoebiasis, vibrio cholera infection, vascular smooth muscle contraction, platelet activation, oxytocin signaling pathway, amino sugar and nucleotide sugar metabolism, and glycolysis/gluconeogenesis in the 3rd-instar nymph stage (Figure 5B). In the adult stage, the DEGs were enriched in metabolic pathways, vascular smooth muscle contraction, protein processing in endoplasmic reticulum, lysosome, carbon metabolism, and amino sugar and nucleotide sugar metabolism (Figure 5C).

### 3.4. Molecular Differentiation of Macropterous and Brachypterous Strains

**Figure 6:**
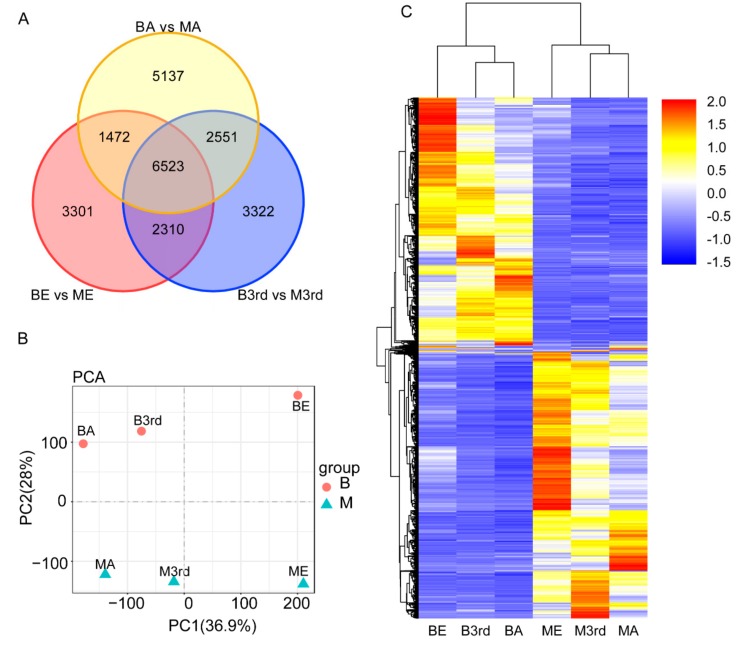
Distribution of DEGs between brachypterous and macropterous strains during egg (ME and BE), nymph (M3rd and B3rd) and adult (MA and BA) stages (**A**), principal component analysis for six samples based on expression levels of 76,742 unigenes (**B**), and hierarchical clustering of six BPH samples from the macropterous and brachypterous strains (ME, M3rd, MA, BE, B3rd, and BA) based on the expression levels of 6523 DEGs (**C**). In the heatmap, red to blue represents the up-regulated to down-regulated expression levels, and *X*-axis is the BPH sample and *Y*-axis represents the DEG.

Wing morph selection caused remarkable changes in the gene expression profiles of BPHs. A total of 6523 DEGs were found to consistently differ between the macropterous and brachypterous strains across all three growth stages: egg, 3rd-instar nymph, and adult (Figure 6A). The six samples from the brachypterous (BE, B3rd, and BA) and macropterous (ME, M3rd, and MA) strains were discriminable based on the first two components that were derived from the FPKM of all 76,742 unigenes (Table S4), and first principle components axis PC1 clearly discriminated based on life stage, whereas the second axis PC2 discriminated based on wing morph (Figure 6B). The eggs, 3rd-instar nymphs, and adults could be clustered well into the brachypterous or macropterous strain while using the expression levels of 6523 DEGs, suggesting significant differences in gene expression levels between the wing strains (Figure 6C).

**Figure 7:**
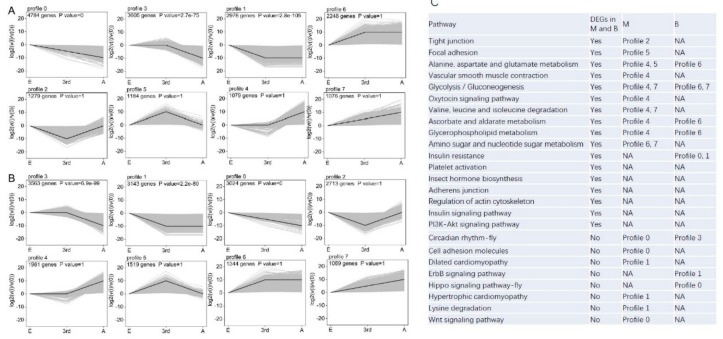
The expression trend profiles (profile 0–7) of genes across egg, nymph, and egg stages in the macropterous (**A**) and brachypterous strains (**B**), and the expression profile of genes belonging to different pathways in the brachypterous and macropterous strains (**C**). NA means no changes in gene expression across the egg, 3rd instar nymph, and adult stages.

The trend analysis for all unigenes among egg, nymph, and adult stages showed that there were three significant trend profiles (profile 0, 1, and 3) out of eight profiles (profile 0–7) in both the macropterous (Figure 7A) and brachypterous (Figure 7B) strains. The general significant expression trends for genes were for a decline from the egg to the adult stage (Figure 7A,B). However, it is important to note that the numbers of genes distributed within each of the three significant trend profiles were different between the macropterous and brachypterous strains. Many genes were distributed to the profile 0 in the brachypterous strain, whereas to profile 3 in the macropterous strain (Figure 7A,B). Moreover, genes that were distributed to the same trend profile in the brachypterous and macropterous strains were usually involved in different KEGG pathways (Figure 7C). The expression profiles for DEGs between the macropterous and brachypterous strains did not change across the three growth stages for six pathways: platelet activation, insect hormone biosynthesis, adherens junction, regulation of actin cytoskeleton, insulin signaling pathway, and PI3K-Akt signaling pathway (e.g., they were constitutively different between strains). In contrast, the DEGs that were enriched in six other pathways did show different expression profiles across the three growth stages for the macropterous and brachypterous strains (tight junction, focal adhesion, vascular smooth muscle contraction, glycolysis/gluconeogenesis, oxytocin signaling pathway, and metabolisms). Meanwhile, the genes in yet a third group of pathways were not differentially expressed between the strains while at the same growth stage, but the expression profiles were different when being considered across the three growth stages (e.g., for circadian rhythm-fly, cell adhesion molecules, hippo signaling pathway-fly, Wnt signaling pathway, etc.). For example, genes in the circadian rhythm-fly pathway belonged to profile 3 in the brachypterous strain, but they belonged to profile 0 in the macropterous strain (Figure 7C).

### 3.5. Expression Levels of Genes in Macropterous and Brachypterous Strains

**Figure 8:**
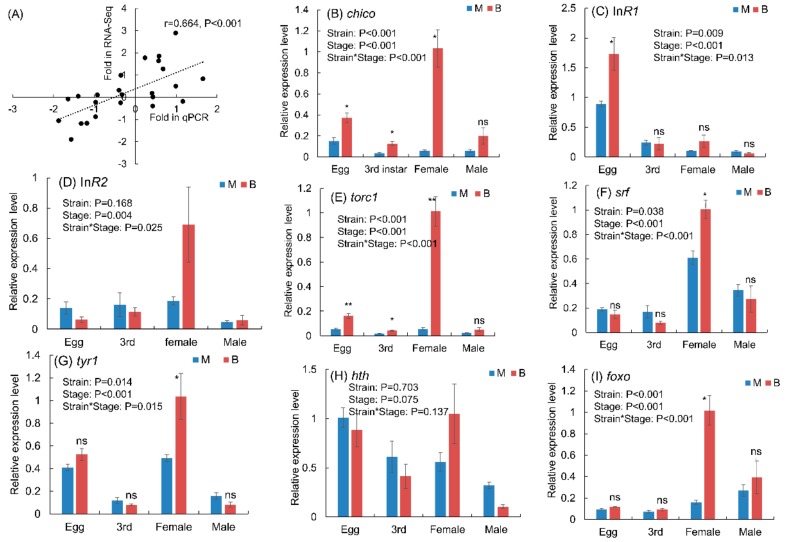
Correlation of fold changes of eight genes between the macropterous strain and brachypterous strain in three growth stages of BPHs measured by RNA-seq with these values measured by qPCR (**A**), and the relative expression levels of eight genes between the brachypterous and macropterous strains among three growth stages (**B**–**I**). M: macropterous strain, B: brachypterous strain. ** and * above the bars mean significant difference at the *p* = 0.01 and *p* = 0.05 level, respectively, and ns means no significant difference between M and B.

The qPCR results showed that fold changes in the expression level of eight selected genes measured by RNA-seq method were significantly related to the values measured by qPCR method in three growth stages (Figure 8A), which suggested the validation of the RNA-seq in this study. The wing-morph strain, growth stage, or the interactions significantly affected the relative expression levels of these six genes *chico*, *InR1*, *torc1*, *srf*, *tyr1*, and *foxo* (Figure 8B,C,E–G,I). But the relative expression level of *hth* was not significantly different between the macropterous and brachypterous strains, and among eggs, 3rd instar nymphs, and adults (Figure 8H). The *InR2* was differentially expressed among three growth stages of BPHs, and the wing morph affected the expression of *InR2* associated with the growth stage (Figure 8D). The expression levels of *chico* and *torc1* were higher in the brachypterous strain than that in the macropterous strain during all three growth stages (Figure 8B,E), and the other genes at least were higher in a specific growth stage (Figure 8C,F,G,I).

## 4. Discussion

Differentiation in the wing morph of insects leads to remarkable changes in the gene expression levels [7,30,33,39]. In this study, we used the pure-bred strains of the short-winged and long-winged BPHs to explore the global changes in gene expression, and found that there were substantial and consistent changes between the macropterous and brachypterous strains across three growth stages, egg, nymph, and adult, but there were also substantial growth stage-dependent changes. These changes in gene expression occurred in the genetic pure-bred strains of wing morphs under a constant environmental condition might result from the genetic basis. Here, we found 15,683 DEGs between adults (including females and males) from the macropterous and brachypterous strains that were twenty times more than the 755 DEGs found between the female macropterous and brachypterous adults measured by Xu et al. [33]. We thought that the number of DEGs between the macropterous and brachypterous pure-bred strains might be larger than the wild line, although there also were 2172 genes with significant expression changes between the macropterous female and male adults [33]. The multigenerational selection for wing morphs may induce more genes to express differentially between the macropterous and brachypterous morphs. Wing selection produced lines with a relatively consistent wing form in BPHs [5,47], which showed the importance of genes in determining wing morphs. From this study, many differentially expressed genes were found between the macropterous and brachypterous strains of BPHs, which suggested that gene expression might determine the wing morphs of BPHs. There were 3882 out of 7909 genes expressed differentially in the functional and histolytic thoracic flight muscles of *Gryllus firmus*, and the fat body of short-winged and long-winged morphs of this insect also exhibited transcriptional differences [48]. These findings can explain well the previous study that the wing morphism of some insects, including rice planthoppers, is under the polygenic control [6,35,49].

Although, in this study, the RNA-seq samples were collected from the 37th and 39th generation of wing selection that were nearly pure-bred lines of the brachypterous and macropterous morphs and the threshold to discriminate DEGs was strict (fold change ≥ 2 and FDR ≤ 0.001), the presence of false-positive DEGs was still not wholly avoid, due to the absence of biological replicates. The high percentages of DEGs in each developmental stage (17–20%) between the brachypterous and macropterous strains may include a few false positive errors. Ideally, the results will be better if it was repeated two additional times with independently inbreed strains, or at least on different generations of the original inbreed strains to account for the variability in the short-winged rate. In this study, we used the FPKM to estimate the expression level of genes, but the Transcripts per Kilobase Million (TPM) might be more reliable than FPKM or RPKM [50]. We reanalyzed the data while using TPM, and DEGs analyzed by TPM were as similar as these by FPKM. A total of 13,606, 14,672, and 15,706 DEGs between the macropterous and brachypterous strains in eggs, 3rd instar nymphs, and adults were found based on TPM, respectively, and it was 13,606, 14,706, and 15,683 based on FPKM (Tables S1–S3). Accordingly, we thought that the methods to estimate the DEGs in this study were suitable and the false-positive error might be low. The method DEseq2, EdgeR, or Limma was recently used for DEGs analysis based on the negative binomial distribution model [32,51], but we performed the analysis with the Poisson distribution method previously used in other studies [52,53], followed the qPCR test for some putative target genes in this study. The old method seemed to be suitable for this RNA-seq data, but the new one might be much better. Therefore, the comparison between different methods of DEG analysis for this RNA-seq data might be considered.

We found in this study that the DEGs between the macropterous and brachypterous strains were mainly distributed to the similar GO terms, such as catalytic activity, binding, metabolic process, and cellular process, and the similar KEGG units, such as global and overview maps, signal transduction, cellular community, endocrine system, and digestive system among the egg, 3rd instar nymph, and adult stages. The results showed that these functional terms and units might be involved in the regulation of wing dimorphism, and these genes related to the cellular and metabolic process, signal transduction, endocrine, and digestion may be the key genetic basis of wing morphs. In aphids, the metabolic and signaling pathways, muscles, and energy production were enriched for DEGs between the winged and wingless morphs [27,28]. Moreover, the significantly enriched GO terms for DEGs between the macropterous and brachypterous BPH strains in eggs were much more than that in nymphs and adults (Table 3). This result showed that genetic differentiations in the molecular level between the macropterous and brachypterous strains were significant in the developmental stage of egg, which suggested the genetic determination of wing morphs in BPHs. Gene profiles of the macropterous and brachypterous strains across developmental stages, to our knowledge, were first identified here, although the genetic basis or inheritance of wing dimorphism in some species of insects, including rice planthoppers, has been found [2,5,35,47,49].

Which are the genes involving in the determinant of wing morphs in insects? This is the key question to address the gene network and molecular mechanism of wing dimorphism. In this study, we found that there might be at least two groups of genes that were differentially expressed between the pure-bred macropterous and brachypterous strains. One group was the constant differentially expressed genes across three growth stages, and here we called ‘global genes’, which were mainly related to the regulation of actin cytoskeleton, focal adhesion, tight junction, and adherens junction. These genes may determine the morphological structure of wings and muscles via mediating various important cellular processes, such as cell structural support, axonal growth, cell movement, cell adhesion, and so on [54,55,56], and they result in the different wing sizes and flight muscle in the macropterous and brachypterous adults [57,58]. We found that the expression levels of *chico* and *torc1* genes in the brachypterous strain were significantly higher than that in the macropterous strain, regardless of the BPH growth stages. The gene *chico* acted autonomously in the control of cell size and organ size [59]. RNAi *chico* in BPHs would result in the short-winged morph [7]. The *torc1* gene regulates multiple cellular processes to control cell growth in response to environmental signals [60]. The genetic information in these global genes might represent the maternal determinant for progeny wing morphs.

The other group genes were differentially expressed between the macropterous and brachypterous strains only in a specific growth stage, named here ‘stage-dependent genes’, which mainly involved in the metabolic pathways. These pathways, the insulin signaling pathway, oxytocin signaling pathway, amino sugar and nucleotide sugar metabolism, carbon metabolism, and glycolysis/gluconeogenesis were significantly enriched with DEGs between the macropterous and brachypterous strains in either eggs or the 3rd instar nymphs. We speculated that the expression of these stage-dependent DEGs might be sensitive to environmental factors, including host quality and crowding, and, consequentially, their expression levels may determine the wing morph. The heritability of wing morphs in many insects was relative low and inconsistent [47,49], which suggested a threshold in the gene expression level might modulate wing forms. A study reported that two insulin receptors (*InR1* and *InR2*) determine the wing morph of the rice planthoppers via regulating the activity of the forkhead transcription factor *foxo*, and the knockdown of *InR1* gene led to the short-winged morph, but the knockdown of *InR2* gene led to the long-winged morph [7]. However, the knockdown of *foxo* directly resulted in the long-winged morph whether the *InR1* and *InR2* were interfered or not [7]. The *srf* transcription factor regulates or promotes tissue growth in *Drosophila*, which is largely based on the overexpression of Pico and Mal reported to increase wing size [61,62]. In this study, we found that the interaction between wing morph strain and developmental stage of BPHs determined the expression levels of *InR1*, *InR2*, *foxo*, and *srf* genes. Environmental conditions, especially host quality, such as glucose concentration, affected the wing morph of BPHs [19]. These environmental conditions may affect insect’s wing morphs via regulating the expression levels of stage-dependent genes. Therefore, environmental changes that only occur in a specific period of insects can result in the wing morph shift between macropterous and brachypterous [21,22,23,24].

**Figure 9:**
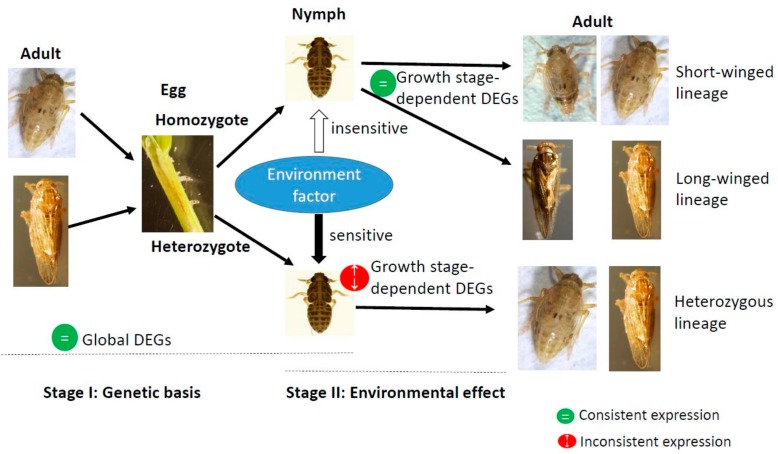
A proposed mode for the determination of BPH’s wing morphs based on genetic and environmental effects. Changes in gene expression levels may determine wing dimorphism.

The nature of the growth stage-dependent genes versus the globally differentially expressed genes between the macropterous and brachypterous strains suggests that the wing morph of BPHs might be regulated via a two-stage genetic path: the first stage is the inheritance from adults that determines the expression of all ‘global genes’, and the other is the environmental effect in nymphs that determines the expression of all ‘stage-dependent genes’ (Figure 9). Among global DEGs, these related to the tight junction, focal adhesion and vascular smooth muscle contraction pathways exhibited different expression trend profiles across the egg, 3rd instar nymph, and adult stages between the macropterous and brachypterous strains, and the others related to the adherens junction and regulation of actin contraction pathways were constantly expressed across the three growth stages of BPH. These global genes may all be intimately involved in the determination of structural foundation of wings, and their expression levels were under genetic control. These growth stage-dependent genes that are related to metabolic pathways, such as insulin signaling pathway, oxytocin signaling pathway, amino sugar and nucleotide sugar metabolism, and carbon metabolism, may finally determine the phenotype of wings via responses to environments in nymphs. We speculate that these genes may be sensitive to environmental conditions only in the heterozygous wing line, but insensitive in the homozygous wing strain, like the global genes. Therefore, the genetic pure-bred line in wing morph can be selected and their offspring’s wing form was not altered by different environmental conditions, such as photoperiod [14,38,47,49]. The genetic and environmental factors may modulate the wing morphs of BPHs via cooperation of two types of genes expressed stage-dependently and stage-independently (Figure 9).

In this study, we found that, after 59 generations of wing morph directional selection, there still were an extremely small amount of short-winged and long-winged individuals that were produced by the macropterous and brachypterous strains, respectively (Figure 1). The result suggests that the pure-bred wing morph are not easy to be fully segregated in BPHs. Mochida (1975) found a BPH strain produced abundant brachypterous adults [63]. Even if the *InR1* or *InR2* was knocked down, not all of the treated BPHs showed a wholly identical wing morph [7]. In field survey, we found that there often were some BPHs on rice that did not grow up to long-winged morphs in fall to migrate, even though they could not overwinter there. These non-migrant BPHs may be genetically homozygous. In a previous study, we found that the macropterous and brachypterous strains in three species of rice planthoppers, *N. lugens*, *Sogatella furcifera*, and *Laodelphax striatellus*, did not response in their wing morphs to photoperiod changes [14]. The pure-bred strains of wing morphs showed different responses to environmental changes, as compared to the natural populations [3,14,18]. These results further support the two-stage genetic mode in the wing dimorphism of BPHs (Figure 9).

## 5. Conclusions

We established nearly pure-bred strains of the long-winged and short-winged morph in BPHs and then analyzed the transcriptome of them. Wing morph selection induced significant changes in gene expression levels between the two strains across three developmental stages (egg, nymph, and adult), and gene expression profiles were different. The significantly enriched pathways for DEGs are either constant across three development stages or stage-dependent. We hypothesized that these constant DEGs might be involved in the solely genetic determination of wing morphs, whereas stage-dependent DEGs might be involved in the wing morph determination by gene and environment interactions. Genes expressed stage-independently and stage-dependently may modulate the wing dimorphism of BPHs.

## Figures and Tables

**Table 1:** Primers for qPCR in this study.

Gene	Primer	Sequence (5′-3′)	Size
Serum response factor (*srf*)	srf-F	GTTCGCCGCCTTCCAAT	145 bp
	srf-R	TGACGCCACCAGTAGCA	
*hth*	hth-F	CAGAAGAAGCGCGGAAT	167 bp
	hth-R	CGCCTCGCATTGATAAAC	
Insulin receptor 1 (*InR1*)	InR1-F	TGCCATCAAGACTGTCAAT	152 bp
	InR1-R	GCTCCATCACCACATAGG	
Insulin receptor 2 (*InR2*)	InR2-F	CTTGCCGAACAGCCTTAC	150 bp
	InR2-R	GGGTCGTTTAGTGGGTCT	
*foxo*	foxo-F	CGCCTGTAACCACCAAA	199 bp
	foxo-R	CTCGTGCTTGATGACCTC	
Target of rapamycin complex 1 (*torc1*)	Torc1-F	GGCTACAGGGATGTCAAAG	162 bp
	Torc1-R	GCATCACCAGCGTTTTATG	
Insulin receptor substrate gene (*chico*)	Chico-F	GCTGGAGTCGTTCTTCTAC	148 bp
	Chico-R	CAAGTCCTTCTGCGAGTTC	
Tyramine receptor 1 (*tyr1*)	tyr1-F	AGGAGGACTCGGTAACTG	182 bp
	tyr1-R	GGTGGCTGGCATTATCAT	
*18S RNA*	18S-F	CGCTACTACCGATTGAA	132 bp
	18S-R	GGAAACCTTGTTACGACTT	

**Table 2:** Output of sequencing and assembly quality for six transcriptomes of the brown planthopper.

Item	Short-Winged Strain			Long-Winged Strain		
	Egg	3rd Instar	Adult	Egg	3rd Instar	Adult
Total raw reads	54,721,540	55,915,498	54,415,048	57,603,588	56,380,930	55,801,748
Total clean reads	51,363,210	52,020,498	51,285,176	54,061,654	52,684,738	52,726,358
Total clean nucleotides (nt)	4,622,688,900	4,681,844,820	4,615,665,840	4,865,548,860	4,741,626,420	4,745,372,220
Q20 percentage	97.87%	97.78%	97.95%	97.83%	97.80%	97.98%
Total number of contig	115,221	104,974	93,064	113,494	101,678	101,760
Total length of contig (nt)	51,970,647	45,085,737	40,546,907	52,387,287	45,157,354	42,697,500
Mean length of contig (nt)	451	429	436	462	444	420
Total number of unigene	72,598	66,433	60,612	71,586	66,103	64,619
Total length of unigene (nt)	67,908,204	52,260,520	46,158,712	68,344,039	52,676,037	48,676,881
Mean length of unigene (nt)	935	787	762	955	797	753
Distinct clusters of unigene	19,705	14,068	11,570	19,325	13,226	12,965
Distinct singletons of unigene	52,893	52,365	49,042	52,261	52,877	51,654

**Table 3:** GO term enrichment for DEGs across egg, 3rd-instar nymph and adult stages between the macropterous and brachypterous strains.

Stage	Type of Ontology	GO Term	Term_ID	Log_10_ Corrected *p*-Value	Uniqueness	Number of DEGs
Egg	Biological process	Cell fate determination	GO:0001709	−6.3675	0.423	27
		Regulation of cell projection organization	GO:0031344	−5.8210	0.498	27
		Morphogenesis of a branching structure	GO:0001763	−5.4486	0.391	25
		Branching morphogenesis of an epithelial tube	GO:0048754	−5.4486	0.323	25
		Regulation of neurogenesis	GO:0050767	−5.3706	0.243	26
		Cell fate commitment	GO:0045165	−3.6383	0.378	37
		Open tracheal system development	GO:0007424	−3.5086	0.389	31
		Respiratory system development	GO:0060541	−3.5086	0.379	31
		Regulation of cell size	GO:0008361	−3.0000	0.607	16
		Negative regulation of cytoskeleton organization	GO:0051494	−2.8894	0.501	17
		Regulation of cell growth	GO:0001558	−2.8894	0.500	17
		Regulation of microtubule-based process	GO:0032886	-2.5272	0.669	15
		Determination of muscle attachment site	GO:0016204	−2.5272	0.453	15
		Taxis	GO:0042330	−2.3242	0.966	33
		Cytoskeleton organization	GO:0007010	−1.9488	0.664	49
		Mushroom body development	GO:0016319	−1.9458	0.426	16
		Negative regulation of cellular component organization	GO:0051129	−1.9374	0.525	18
		Animal organ morphogenesis	GO:0009887	−1.8901	0.312	59
		Regulation of multicellular organismal process	GO:0051239	−1.8380	0.392	30
		Regulation of developmental process	GO:0050793	−1.6552	0.295	35
		Regulation of protein complex disassembly	GO:0043244	−1.5176	0.476	15
		Tube development	GO:0035295	−1.3578	0.353	49
	Cellular component	Site of polarized growth	GO:0030427	−4.0414	0.833	19
		Growth cone	GO:0030426	−4.0414	0.770	19
		Astral microtubule	GO:0000235	−3.6990	0.577	15
		Aster	GO:0005818	−3.6990	0.621	15
		Spindle microtubule	GO:0005876	−3.6990	0.592	15
		Spindle	GO:0005819	−3.5850	0.607	18
		Adherens junction	GO:0005912	−3.1938	0.692	19
		Fusome	GO:0045169	−3.1427	0.759	15
		Cytoplasmic microtubule	GO:0005881	−3.1427	0.566	15
		Neuron part	GO:0097458	−2.1506	0.828	24
		Apical part of cell	GO:0045177	−1.8884	0.834	19
		Apicolateral plasma membrane	GO:0016327	−1.7804	0.703	10
		Subapical complex	GO:0035003	−1.7804	0.580	10
		Cell cortex part	GO:0044448	−1.7352	0.572	11
	Molecular function	Microtubule binding	GO:0008017	−2.4473	0.633	15
		Phosphate transmembrane transporter activity	GO:1901677	−2.0022	0.584	14
		Organophosphate ester transmembrane transporter activity	GO:0015605	−2.0022	0.521	14
		Carbohydrate kinase activity	GO:0019200	−1.8655	0.705	10
		Hexokinase activity	GO:0004396	−1.6906	0.706	8
		Organic phosphonate transmembrane transporter activity	GO:0015604	−1.6029	0.597	12
		Catalytic activity	GO:0003824	−1.4090	0.926	689
		Transferase activity	GO:0016740	−1.4046	0.813	260
3rd instar nymph	Biological process	Metabolic process	GO:0008152	−1.8339	1	593
	Cellular component	No significantly enriched terms				
	Molecular function	Catalytic activity	GO:0003824	−5.3363	0.889	778
		Antioxidant activity	GO:0016209	−4.6536	0.696	30
		Oxidoreductase activity, acting on peroxide as acceptor	GO:0016684	−5.5171	0.588	30
		Transaminase activity	GO:0008483	−2.1337	0.563	19
		Hexokinase activity	GO:0004396	−1.3505	0.578	8
		Transferase activity, transferring nitrogenous groups	GO:0016769	−2.1337	0.563	19
		Peroxidase activity	GO:0004601	−3.6198	0.588	24
Adult	Biological process	Metabolic process	GO:0008152	−2.5045	0.568	658
		Oxidation-reduction process	GO:0055114	−4.3915	0.023	132
	Cellular component	Cytoplasm	GO:0005737	−1.7203	1	242
	Molecular function	Antioxidant activity	GO:0016209	−1.6189	0.254	27
		Oxidoreductase activity, acting on peroxide as acceptor	GO:0016684	−2.2262	0.233	27
		Oxidoreductase activity	GO:0016491	−3.699	0.297	170

## Supplementary Materials

The following are available online at https://www.mdpi.com/2073-4425/11/1/19/s1, Table S1: The screened DEGs (FC ≥ 2 and FDR ≤ 0.001) in eggs between the long-winged and short-winged strains (xls), Table S2: The screened DEGs (FC ≥ 2 and FDR ≤ 0.001) in the third instar nymphs between the long-winged and short-winged strains (xls), Table S3: The screened DEGs (FC ≥ 2 and FDR ≤ 0.001) in adults between the long-winged and short-winged strains (xls). Table S4: The FPKM of 76,742 unigenes in all six samples.
